# Effects of Different Spatial Extents of Occurrence Data on Biomod2-Based Species Distribution Modeling and Prediction: A Case Study of the Potential Distribution of *Spodoptera frugiperda* in China

**DOI:** 10.3390/insects17080843

**Published:** 2026-08-14

**Authors:** Junke Nan, Maofa Yang, Zhipeng He, Baoqian Lyu, Rulin Wang, Danping Xu, Zhihang Zhuo

**Affiliations:** 1 College of Ecology and Agriculture, Sichuan Minzu College, Kangding 626001, China; junkenan@163.com; 2 College of Tobacco Science, Guizhou University, Guiyang 550025, China; 3 College of Life Science, China West Normal University, Nanchong 637002, China; 4 Environment and Plant Protection Institute, Chinese Academy of Tropical Agricultural Sciences, Haikou 571101, China; 5 Sichuan Province Agro-Meteorological Center, Chengdu 610071, China; 6 Environment-Friendly and Efficient Water-Saving Technology and Equipment for Hilly Agriculture Key Laboratory of Sichuan Province, Chengdu 610066, China

**Keywords:** *Spodoptera frugiperda*, climate change, Biomod2, ArcGIS, invasive pest, ensemble model

## Abstract

Climate change is reshaping species distributions worldwide, making reliable prediction of invasive species increasingly important for ecological risk assessment and pest management. However, species distribution models (SDMs) calibrated with regional occurrence records may underestimate potential suitable habitats because of niche truncation. Here, we evaluated the effects of occurrence data extent on SDM predictions using the globally invasive agricultural pest *Spodoptera frugiperda* (J. E. Smith, 1797) (Lepidoptera: Noctuidae). Global occurrence records (1763 records) and China-specific occurrence records (946 records) were combined with climatic, topographic, and vegetation variables to construct ensemble SDMs using the Biomod2 platform under current and future climate scenarios (SSP1-2.6, SSP2-4.5, and SSP5-8.5). Model performance, environmental variable contributions, habitat suitability patterns, and centroid shifts were compared between the two datasets. The global model showed excellent predictive performance (True Skill Statistic (TSS) = 0.851, Receiver Operating Characteristic (ROC) = 0.977) with balanced contributions from multiple environmental variables, whereas the China model achieved only acceptable performance (TSS = 0.690, ROC = 0.905) and relied predominantly on Bio06. Both models consistently identified southern China and the Huang-Huai Plain as the core highly suitable regions. Future projections revealed under higher emission scenarios (SSP5-8.5), particularly toward northern and western China. Centroid analyses further indicated consistent northwestward shifts under higher emission scenarios (SSP5-8.5), whereas low-emission scenarios produced less directional and more variable centroid movements. These results demonstrate that the spatial extent of occurrence records exerts a greater influence on SDM predictions than differences among modeling algorithms. Although models of China may underestimate invasion risk because invasive populations have not reached ecological equilibrium, the spatial stability of highly suitable habitats and centroid migration direction provide robust indicators for cross-model comparisons. This study provides methodological guidance for occurrence data selection and improves invasion risk assessment and management of *S. frugiperda* under climate change.

## Simple Summary

Accurate prediction of invasive species distributions is essential for improving early warning and management under climate change. Species distribution models (SDMs) are commonly developed using occurrence records collected within a target region, but such data may not fully represent the environmental conditions suitable for invasive species that are still expanding their ranges. Using the fall armyworm (*Spodoptera frugiperda* (J. E. Smith, 1797) (Lepidoptera: Noctuidae)) as a case study, we compared SDMs built with global occurrence records and those based only on records from China. Models calibrated with global data provided more reliable predictions and captured a broader range of environmental conditions, whereas models of China tended to underestimate potential suitable habitats because of niche truncation. Despite these differences, both models consistently identified southern China and the Huang-Huai Plain as the major high-risk areas and predicted a northwestward shift in suitable habitats under future climate change. Our findings emphasize that the spatial extent of occurrence data is a critical factor influencing SDM predictions and should be carefully considered when assessing invasion risks and planning pest management strategies.

## 1. Introduction

Global climate is changing at an unprecedented rate [1]. In response to such rapid environmental changes, species are shifting their geographic ranges to track suitable environmental conditions across diverse taxonomic groups worldwide [2,3,4,5]. However, species’ spatial responses to climate change are neither uniform nor synchronous, posing significant challenges for ecosystem functioning, biodiversity conservation, and pest management [6]. Consequently, increasing efforts have been devoted in recent years to quantifying and predicting changes in species distributions within the fields of ecology and biogeography, particularly under ongoing and future environmental changes [7].

With the development of modeling approaches, the analytical framework of species distribution modeling has been continuously improved. From traditional statistical regression techniques to advanced machine learning algorithms, and from models based on single climatic variables to multidimensional frameworks incorporating soil properties, topography, and anthropogenic factors, researchers have progressively refined modeling strategies to better characterize species–environment relationships [8]. Among these approaches, species distribution models (SDMs) have become fundamental tools for predicting potential species distributions. SDMs integrate species occurrence records with environmental variables to establish relationships between environmental conditions and species occurrence probabilities, habitat suitability, or potential geographic distributions, thereby enabling predictions of species distributions under current and future environmental scenarios [9]. A wide range of SDM algorithms has been developed, including regression-based, machine learning, and maximum entropy approaches [10]. Because different algorithms vary in their assumptions, assumptions and predictive performance, no single modeling approach can universally provide optimal predictions. To address this limitation, ensemble modeling frameworks have been developed. To address this limitation, ensemble modeling frameworks such as Biomod2 have been developed, which combine multiple high-performing models to reduce algorithm-specific uncertainty and improve prediction robustness [11].

Current species distribution models (SDMs) are primarily developed by integrating species occurrence records with multiple environmental variables, including climatic, topographic, and soil-related factors [12,13]. Within this framework, the selection of the modeling extent is often determined by specific research objectives. However, regional-scale modeling approaches involve both potential limitations and practical advantages [14,15]. A major limitation of regional-scale models is the potential occurrence of niche truncation. For invasive or widely distributed species, occurrence records restricted to a target region may represent only a subset of the species’ actual geographic range. Consequently, the environmental space available for model calibration may fail to capture the full realized or fundamental niche of the species. Even when models achieve high statistical performance, insufficient representation of critical environmental gradients may lead to biased predictions and underestimate potential suitable areas under future climate change or range expansion scenarios [15]. Nevertheless, regional-scale modeling can be appropriate under specific circumstances. For species with distributions restricted to a particular geographic region, models based on regional occurrence data and environmental variables may better capture local species–environment relationships and provide higher spatial resolution predictions. For regional conservation planning, habitat suitability assessments, and localized invasion risk evaluations [14].

*Spodoptera frugiperda* (J. E. Smith, 1797) (Lepidoptera: Noctuidae), commonly known as the fall armyworm, is a globally important polyphagous agricultural pest native to the Americas [16]. Its larvae can infest over 353 plant species, including maize, cotton, sorghum, and rice, causing substantial yield losses [17,18,19,20]. Since its first detection in West Africa in 2016, *S. frugiperda* has rapidly expanded across Africa, Asia, and Oceania, and has recently been reported in Europe, becoming a major invasive pest of global concern [20,21,22]. In China, it was first detected in Yunnan Province in 2019 and has since spread rapidly to numerous provinces, posing a persistent threat to major crops [19,20]. Most previous SDM studies on *S. frugiperda* have relied on regional-scale occurrence records, whereas global-scale predictions remain limited [15,23]. A critical consideration in designing this comparison is the choice of regional extent. China was selected as the regional comparison unit for three reasons. First, China is one of the world’s largest maize- and rice-producing countries, making it a natural and policy-relevant unit for invasion risk assessment [19,24,25]. Second, *S. frugiperda* is still undergoing rapid range expansion in China and has likely not yet reached ecological equilibrium with local environments, making it an ideal case for testing niche truncation [19,20,26,27]. Third, China’s national administrative boundaries provide a clearly defined and data-rich region with relatively uniform monitoring efforts, essential for a robust comparison against the global model [1,28]. Biogeographic or climatic zones, in contrast, would pool data from multiple countries with vastly different invasion histories and monitoring standards, introducing confounding factors [29,30]. Therefore, China represents a meaningful and methodologically tractable case for evaluating the influence of data spatial extent on SDM predictions [7,8]. This study developed SDMs using both global-scale and China-scale occurrence datasets to evaluate how data extent influences model performance, variable contributions, and habitat suitability prediction.

Study hypothesized that: (1) global-scale occurrence data yield higher model performance than China-only data; (2) the China model exhibits stronger dependence on fewer environmental variables and predicts more limited suitable habitats due to niche truncation; and (3) despite these differences, both models identify consistent core suitable areas and predict a northwestward centroid shift under future warming. By assessing the applicability and limitations of regional-scale models in global invasion risk prediction, this study aims to provide scientific support for risk forecasting, targeted monitoring, and early management of *S. frugiperda*.

## 2. Materials and Methods

### 2.1. Study Design and Modeling Workflows

To evaluate the effects of occurrence data spatial extent on SDM predictions, we constructed two independent ensemble models using Biomod2. The Global model was calibrated with worldwide occurrence records (n = 1763) and environmental predictors selected from global-scale correlation analyses. The China model was calibrated with records restricted to China (n = 946) and predictors selected independently based on correlations within China. Both models followed identical procedures for pseudo-absence generation, data partitioning (75%/25%), replication (10 runs), algorithm selection, performance criteria (TSS ≥ 0.65, ROC-AUC ≥ 0.90), and ensemble construction (weighted mean). This parallel design isolates the influence of data extent on model outcomes, while acknowledging that retained predictors may differ due to region-specific collinearity structures [31]. Three comparisons were performed: (1) model performance (ROC-AUC, TSS, Kappa); (2) environmental variable contributions; and (3) habitat suitability patterns (spatial mapping, area calculations, centroid shifts) under current and future scenarios.

### 2.2. Occurrence Data Sources

**Figure 1:**
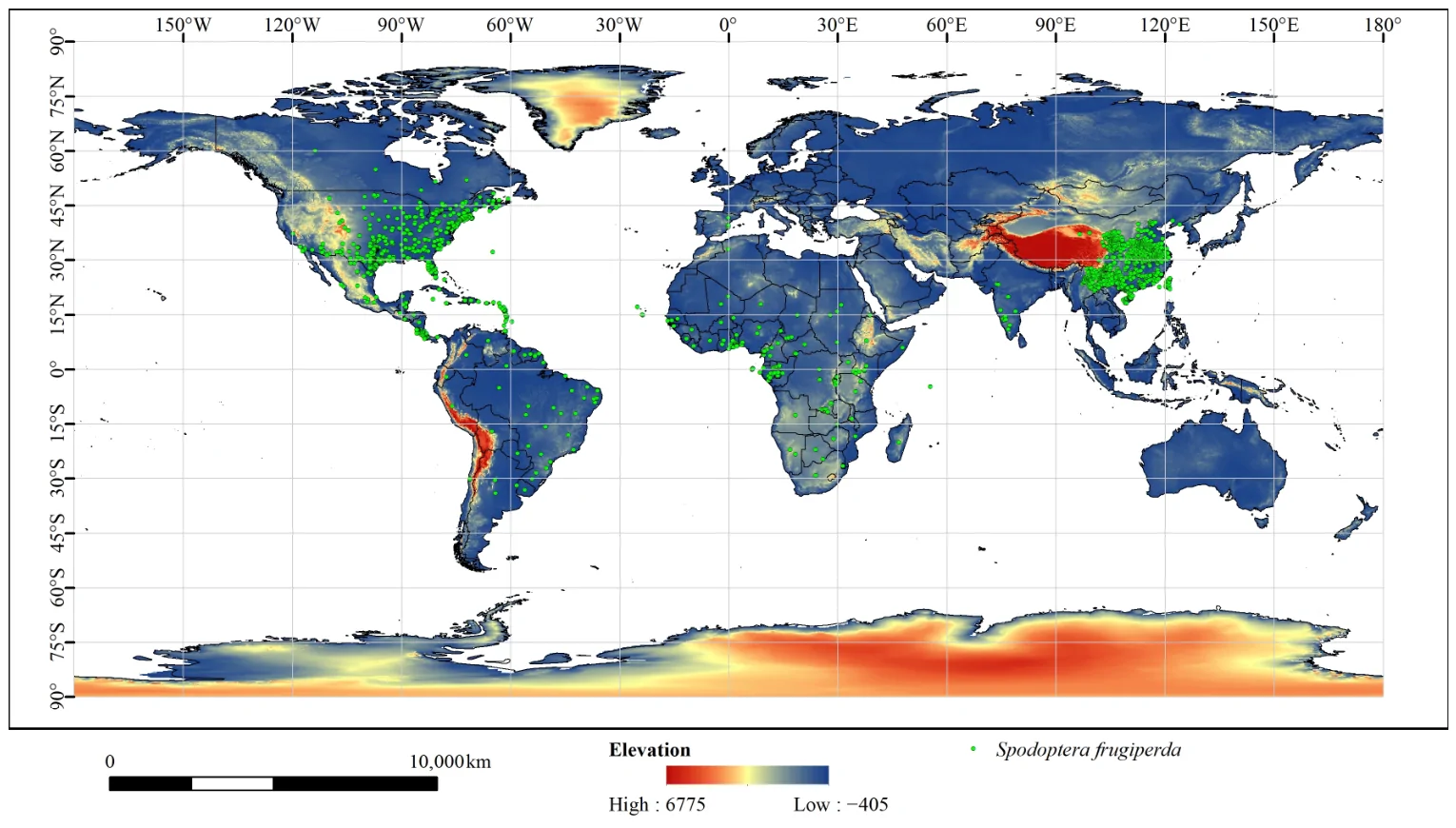
Global occurrence records of *Spodoptera frugiperda* (color gradient from blue to red represents increasing elevation, Green dots in the figure represent occurrence records of *S. frugiperda*).

Occurrence records of S. frugiperda were compiled from GBIF (https://doi.org/10.15468/dl.mvr2h5, accessed on 20 June 2026), the published literature, and field surveys. Records with uncertain coordinates, duplicates, or non-natural occurrences (e.g., artificial introductions without established populations) were removed. Spatial thinning was performed using ENMTools (V1.1.2) at 2.5 km × 2.5 km to reduce sampling bias and autocorrelation, balancing data retention with the species’ high dispersal capacity and the ~1 km environmental resolution. After filtering, 1763 valid records were retained for the Global model, of which 946 were located within China and used exclusively for the China model (Figure 1).

### 2.3. Environmental Variables and Data Preparation

Environmental predictors were drawn from 23 variables: 19 bioclimatic variables (Bio1–Bio19), three topographic variables (aspect, elevation, slope), and one vegetation variable—Percent Tree Cover (gm_ve)—representing tree canopy density as a percentage of ground surface area (MODIS/Terra, Global Map Global Version 2, 1 km). Bioclimatic and topographic layers were obtained from WorldClim 2.1 [28]. All layers were resampled to a unified 30 arc-second (~1 km) resolution with WGS-84 projection using ArcGIS 10.8. Future climate projections (2050s, 2070s, 2090s) were derived from BCC-CSM2-MR under SSP1-2.6, SSP2-4.5, and SSP5-8.5 within CMIP6.

To reduce multicollinearity, Pearson correlation analysis was performed (Supplementary Figure S1). When |r| > 0.8, redundant predictors were removed based on variable importance scores (permutation-based in Biomod2, calculated as 1—Pearson correlation between reference and shuffled predictions), ecological relevance, and practical considerations [32]. Following correlation analysis and model evaluation, 13 variables were retained for the global-scale model and 10 for the China-scale model (Table 1).

### 2.4. Environmental Variable Selection and Model Construction

Species distribution models were developed using Biomod2. Pseudo-absence points (n = 10,000) were randomly generated [33]. Model parameters were optimized using the biomodtuning function to mitigate overfitting. Each model was run with 10 replicates using 75% of records for training and 25% for evaluation, with presence and pseudo-absence equally weighted. Model performance was evaluated using TSS, ROC-AUC, and Kappa. Only algorithms with TSS ≥ 0.65 and ROC-AUC ≥ 0.90 were retained for ensemble modeling. Kappa was not used as a selection criterion due to its sensitivity to prevalence, which can bias comparisons when prevalence differs between calibration extents [31]. High-performing algorithms were combined using the weighted mean approach in Biomod2.

### 2.5. Habitat Suitability Classification, Range Change Analysis, and Centroid Shift Analysis

Suitability thresholds were determined using the maximizing TSS criterion, which balances omission and commission errors and is independent of prevalence [34,35]. This yielded threshold values of 0.25 for the Global model and 0.27 for the China model. Suitable areas were further divided into three classes (low, moderate, high) using equal-interval division based on normalized suitability scores [8,26]. This classification was applied consistently across both models and all climate scenarios.

To quantify future changes, raster layers for the 2050s, 2070s, and 2090s were compared with the current scenario. The vegetation variable (gm_ve) was held constant at current conditions, as future projections at the required resolution are unavailable from CMIP6 [26]. Centroid analysis was conducted using SDMtoolbox v2.6 to calculate the spatial centroid of suitable habitats under different climate scenarios; the direction and distance of centroid shifts were visualized to characterize range displacement.

## 3. Results

### 3.1. Model Performance Evaluation and Key Environmental Predictors

The results revealed substantial differences in predictive performance between models based on global-scale and China-scale occurrence datasets. Models calibrated with global occurrence records generally showed higher predictive performance than those calibrated with China-only records. For the global dataset, XGBoost, MaxEnt, and MaxNet exhibited the highest performance, with mean TSS values above 0.75 and ROC-AUC values above 0.94. GBM, GLM, and MARS also performed well, with mean TSS values exceeding 0.70 and ROC-AUC values above 0.90. CTA, GAM, and FDA showed moderate performance, with TSS values above 0.65 but ROC-AUC values below 0.90. ANN and SRE showed relatively poor performance, with ANN having a mean TSS below 0.60 and SRE showing the lowest predictive ability (Table 2). For models based on China-scale occurrence records, overall predictive performance was reduced. XGBoost achieved the highest performance, with mean TSS values above 0.70 and ROC-AUC values above 0.90. XGBoost, MaxEnt, MaxNet, GBM, GLM, and MARS met the criteria for acceptable performance (TSS ≥ 0.65 and ROC-AUC ≥ 0.90). FDA and GAM showed moderate performance, whereas ANN, CTA, and SRE exhibited lower predictive accuracy. Among these algorithms, SRE showed the poorest performance, with mean TSS values below 0.60 and ROC-AUC values below 0.80 (Table 2).

Notably, the RF algorithm exhibited near-perfect calibration scores (TSS = 1.000, ROC = 1.000) for both datasets, which is a typical indicator of overfitting due to its high model complexity and tendency to memorize training patterns rather than learning generalizable ecological relationships. Given that the primary objective of our study was to produce transferable predictions under future climate scenarios, RF was excluded from the ensemble modeling process. Only algorithms with acceptable or higher predictive performance were retained for ensemble model construction. For the model based on global occurrence data, six high-performing algorithms were selected and combined using a weighted ensemble approach. The resulting ensemble model exhibited excellent predictive performance, with a TSS value of 0.851 and a ROC-AUC value of 0.977, indicating strong model reliability. For the model based on China occurrence data, six algorithms were also selected for weighted ensemble modeling. The resulting ensemble model showed good predictive performance, with a TSS value of 0.690 and a ROC-AUC value of 0.905, suggesting that the model provided reliable predictions of the potential distribution of *Spodoptera frugiperda* in China. Notably, for the China dataset, the ensemble model underperformed the best individual algorithm, XGBoost (TSS = 0.787), indicating that model averaging does not guarantee improved performance when training data are ecologically restricted.

The environmental variables selected for ensemble modeling differed between the models based on global occurrence data and China occurrence data. For the ensemble model constructed using global occurrence records, the contributions of environmental variables were relatively balanced. Among the 13 selected predictors, 10 variables contributed more than 1% to model predictions. Four variables, namely Bio08, Bio16, Bio09, and Bio03, each contributed more than 10% and were identified as the key environmental predictors. Together, these four variables accounted for 80.4% of the total contribution, with Bio08 showing the highest individual contribution (31.66%) (Supplementary Table S2). In contrast, the ensemble model based on China occurrence records showed a strong dependence on a limited number of environmental variables. Only four predictors, including Bio04, Bio06, elevation (elev), and vegetation coverage (gm_ve), contributed more than 1%, with contribution rates of 2.893%, 84.383%, 1.239%, and 8.686%, respectively. Among these variables, Bio06 alone contributed 84.383%, substantially exceeding the contributions of all other predictors, indicating that the China-scale model was strongly dominated by Bio06 (Supplementary Table S2).

### 3.2. Potential Distribution of S. frugiperda Under the Current Climate Scenario

**Figure 2:**
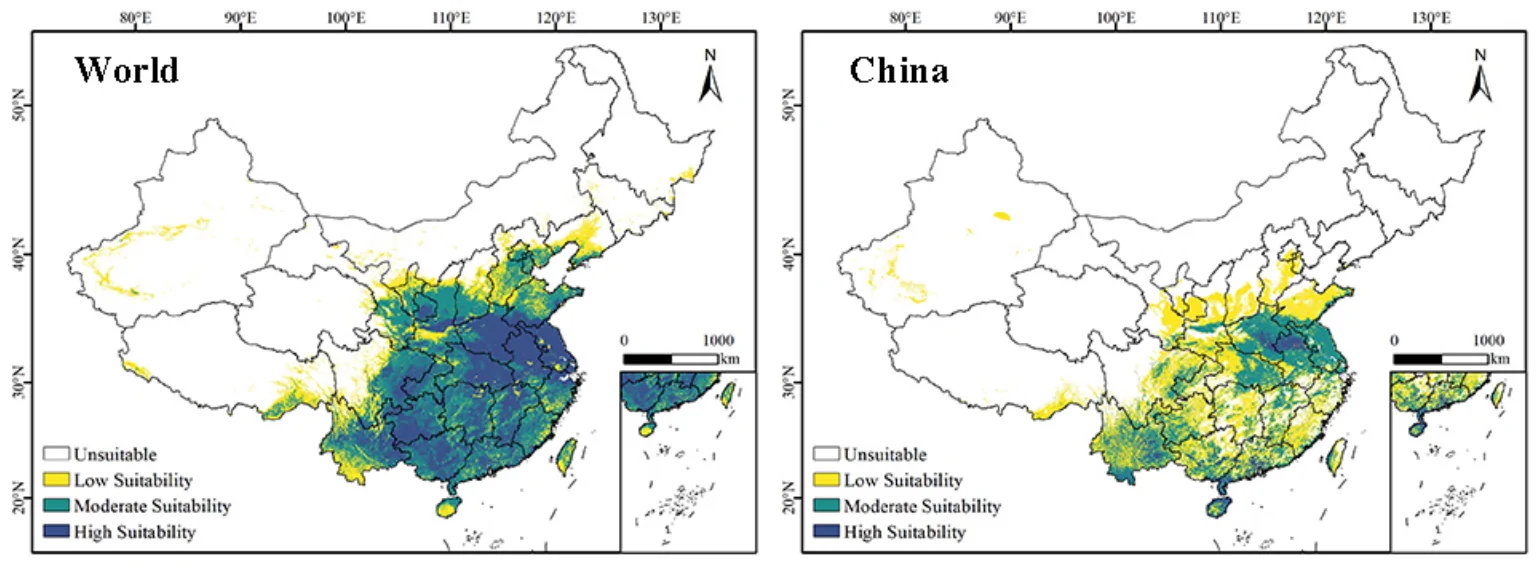
Potential distribution pattern of *Spodoptera frugiperda* under the current climate scenario.

According to the ensemble model predictions, under the current climate scenario, the model based on global occurrence data identified approximately 3.46231 × 10^6^ km^2^ of China as potentially suitable habitat for *S. frugiperda* (Table 3). Among these suitable areas, highly suitable habitats accounted for 34.06% and were mainly distributed across most parts of southern China and the Huang-Huai Plain. Particularly, Hunan, Henan, Anhui, and Jiangsu provinces exhibited clear spatial aggregation of highly suitable habitats. Moderate- and low-suitability areas accounted for 42.91% and 23.03% of the total suitable area, respectively. These areas were primarily distributed across the Yangtze River Basin, Jianghuai region, and extensive areas north of the Lingnan–Huaihe region. A small proportion of suitable habitats was also predicted in southern Tibet, western Xinjiang, and eastern Heilongjiang. Overall, the global occurrence-based model revealed a spatial gradient pattern characterized by highly suitable habitats as the core areas, moderate-suitability habitats as transitional zones, and low-suitability habitats extending toward peripheral regions. In comparison, the ensemble model based on China occurrence data predicted approximately 2.40908 × 10^6^ km^2^ of potentially suitable habitat under the current climate scenario (Table 3), which was considerably smaller than the area predicted by the global occurrence-based model. Highly suitable, moderately suitable, and low-suitability habitats accounted for 56.29%, 38.39%, and 5.31% of the total suitable area, respectively. Highly suitable habitats were mainly concentrated along the border between Henan and Anhui provinces, as well as in southern Yunnan, Guangdong, Guangxi, and Hainan Island, showing a relatively fragmented distribution pattern. Moderate- and low-suitability habitats were mainly distributed in central-southern China, eastern China, and the eastern part of northwestern China. Similar to the global model, these areas exhibited a spatial expansion pattern from highly suitable cores to surrounding regions. However, suitable habitats in central-southern and eastern China were mainly distributed as continuous patches, whereas those in southern central China and northwestern China occurred primarily as scattered patches (Figure 2).

### 3.3. Potential Distribution Changes in S. frugiperda Under Future Climate Scenario

**Figure 3:**
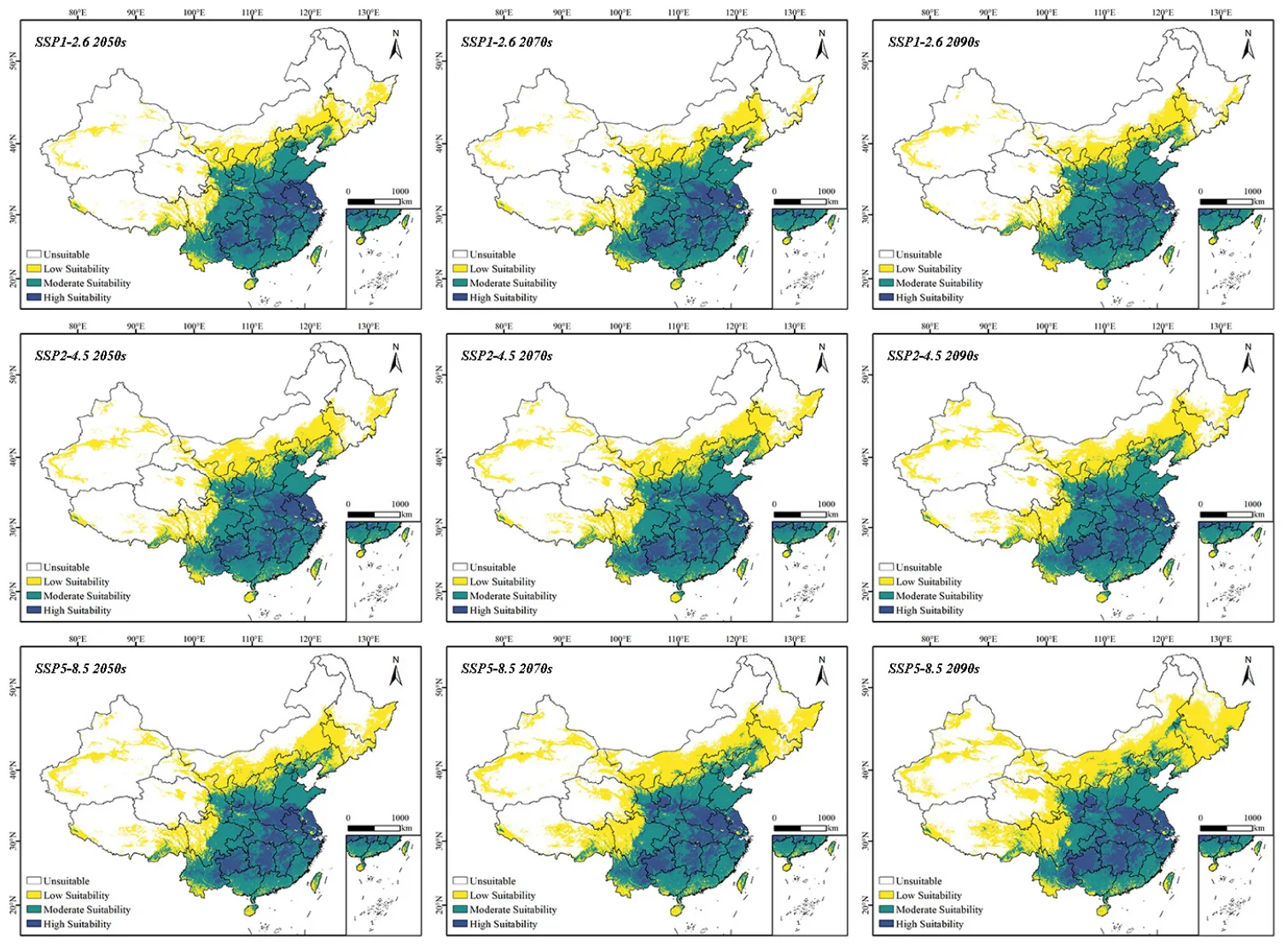
Predicted potential distribution of *Spodoptera frugiperda* in China under future climate scenarios based on the model constructed using global occurrence data.

For the Global model, suitable habitat expanded under all future scenarios, though patterns differed among climate pathways. Under the SSP1-2.6 scenario, the suitable habitat area of *S. frugiperda* showed a nonlinear temporal pattern, with an initial increase followed by a decrease and subsequent recovery; while highly suitable areas declined continuously. Under SSP2-4.5, suitable habitats expanded northward, mainly driven by increases in low-suitability areas; Under SSP5-8.5, the strongest expansion occurred, with suitable habitats extending substantially northward and westward; by the 2090s, total suitable area increased by nearly 80% compared with the current scenario, reaching 6.183 × 10^6^ km^2^ (64.33% of China’s land area), primarily due to the outward expansion of low-suitability habitats (Figure 3).

**Figure 4:**
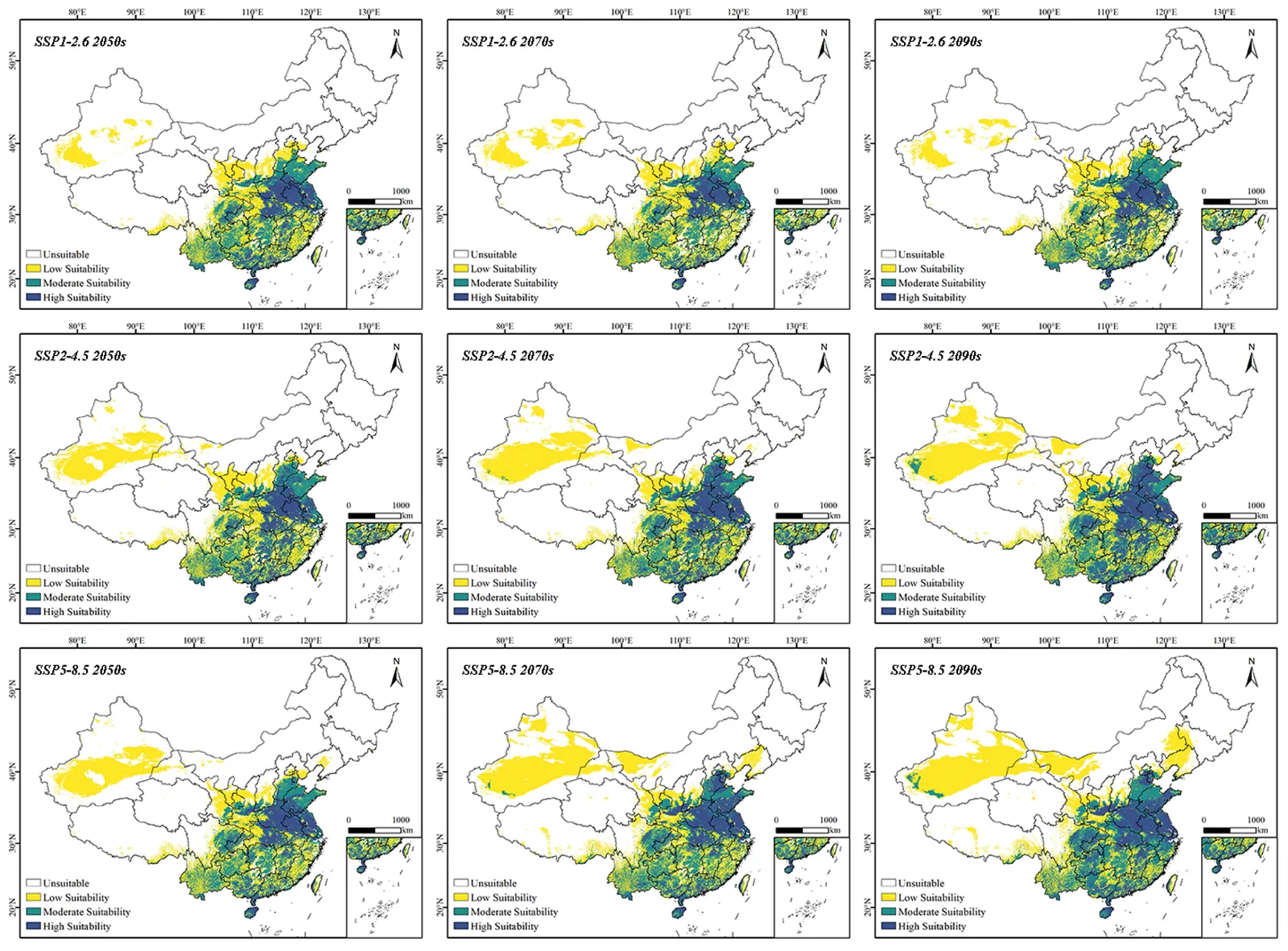
Predicted potential distribution of *Spodoptera frugiperda* in China under future climate scenarios based on the model constructed using China occurrence data.

For the China model, suitable habitats also expanded under all scenarios. Under SSP1-2.6, total area remained relatively stable, but suitability levels improved in some regions, increasing highly suitable habitats. Under SSP2-4.5, suitable areas expanded westward, with low-suitability habitats extending outward and some areas transitioning to higher suitability levels. Under SSP5-8.5, the most pronounced northward expansion occurred, by the 2090s, total suitable area increased by more than 85%, reaching 4.467 × 10^6^ km^2^ (46.48% of China’s land area) with increases across all suitability classes (Figure 4). Given that our primary objective is to compare the Global models and models of China rather than to interpret fine-scale differences among scenarios, we focus on overall patterns of expansion or stability rather than on minor quantitative variations between individual scenarios.

### 3.4. Future Habitat Changes and Centroid Shifts

**Figure 5:**
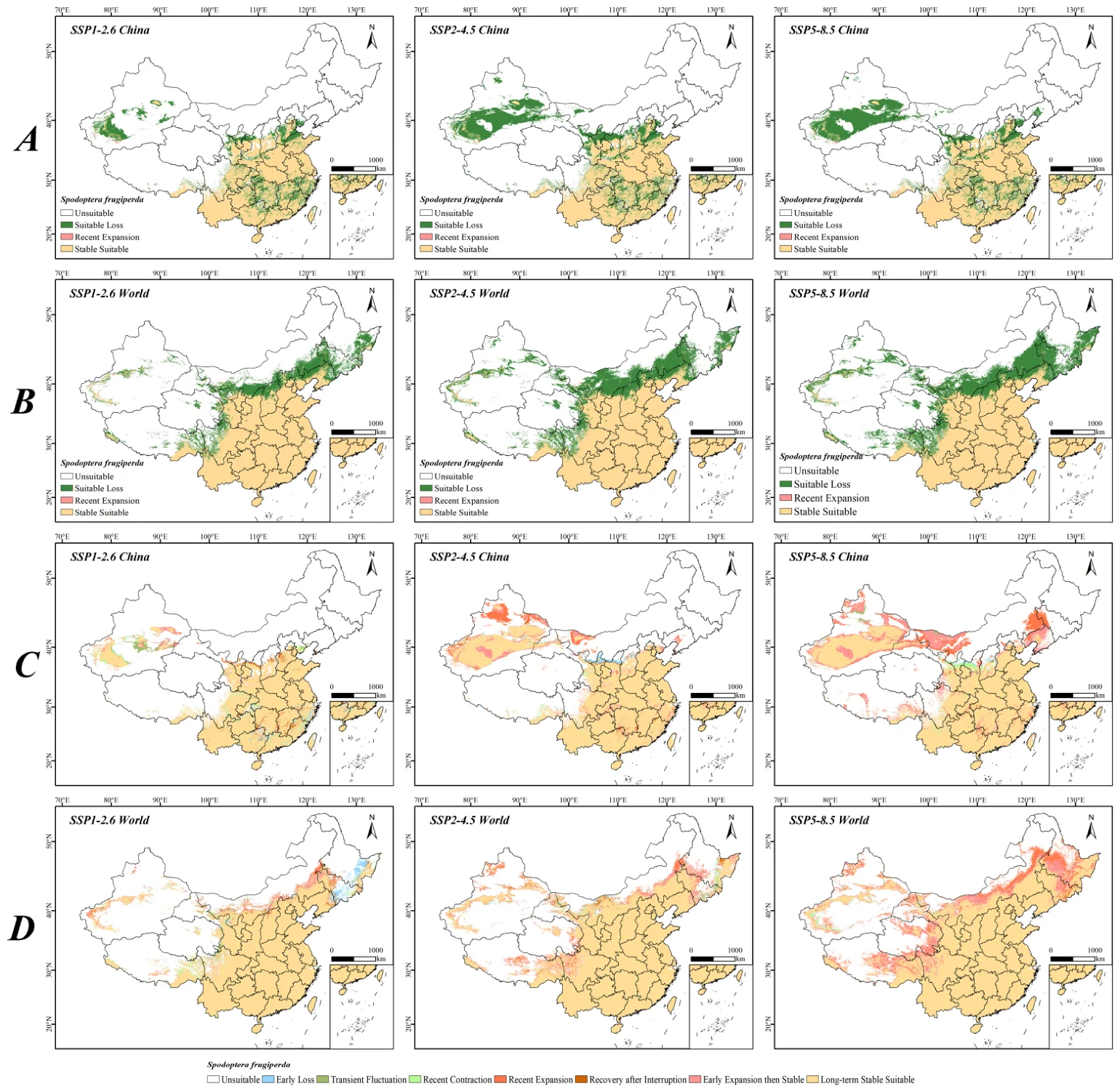
Changes in suitable habitats predicted by models constructed using different occurrence datasets under different future climate scenarios. (**A**): Changes in suitable habitats from the current climate scenario to the 2050s based on the model constructed using China occurrence data; (**B**): changes in suitable habitats from the current climate scenario to the 2050s based on the model constructed using global occurrence data; (**C**): subsequent changes in suitable habitats under different future climate scenarios based on the model constructed using China occurrence data; (**D**): subsequent changes in suitable habitats under different future climate scenarios based on the model constructed using global occurrence data. Panels (**C**,**D**) share the same bottom legend).

Spatial change analyses based on different climate scenarios revealed that, regardless of the occurrence dataset used for model calibration, the suitable habitat of *S. frugiperda* showed a certain degree of reduction from the current climate scenario to the 2050s, particularly in western and northern China. The reduction was most pronounced in the global occurrence-based model under the SSP5-8.5 scenario, whereas the China occurrence-based model under the SSP1-2.6 scenario showed the smallest magnitude of change. Further analyses of habitat dynamics under different future periods and climate scenarios indicated distinct patterns of suitable habitat change among emission pathways. For the China occurrence-based model, suitable habitats remained relatively stable under the SSP1-2.6 scenario, with limited spatial changes. Under the SSP2-4.5 scenario, suitable habitats mainly expanded in western China, while a small proportion of suitable areas was lost in the northern margins of relatively stable suitable regions. Under the SSP5-8.5 scenario, the spatial change pattern was generally consistent with that under SSP2-4.5 but occurred with greater magnitude, indicating stronger habitat redistribution under higher greenhouse gas emissions. For the global occurrence-based model, habitat change patterns were generally similar among different climate scenarios but varied in expansion intensity. Under the SSP1-2.6 scenario, suitable habitats mainly expanded toward northern China, accompanied by a noticeable reduction in parts of northeastern China. Under the SSP2-4.5 scenario, suitable habitats further expanded into western and northern China, while reductions continued in northeastern regions. Under the SSP5-8.5 scenario, suitable habitats exhibited the strongest outward expansion, resulting in a substantial enlargement of the potential distribution range of *S. frugiperda* (Figure 5).

**Figure 6:**
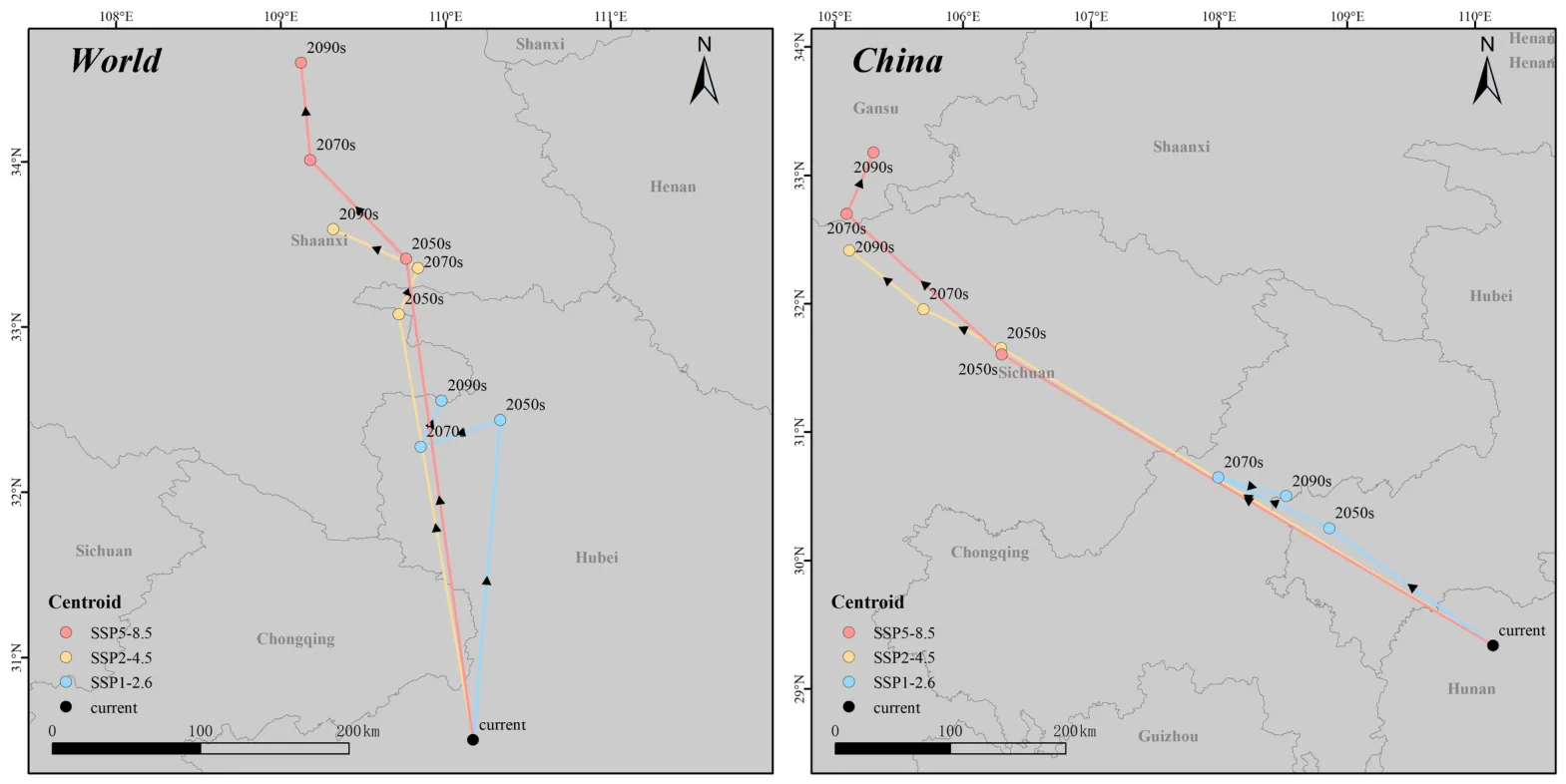
Centroid shifts in the predicted potential distribution of *Spodoptera frugiperda* based on models constructed using different occurrence datasets.

Distribution centroids of suitable habitats under different climate scenarios were calculated and visualized using SDMtoolbox (Figure 6). This tool calculates the area-weighted mean center of all suitable habitat cells, where each cell is weighted by its area. For the global model, the current centroid was in Hebei Province. Under SSP1-2.6, it shifted northeastward, then southwestward, and finally northeastward to the border between Shanxi and Shaanxi. Under SSP2-4.5, it moved northwestward, then northeastward, and finally northwestward to central Shaanxi. Under SSP5-8.5, it continuously shifted northwestward to northern Shaanxi. For the China model, the current centroid was in Henan Province. Under SSP1-2.6, it shifted northwestward twice and finally southeastward to Chongqing. Under SSP2-4.5, it showed a continuous westward shift to northern Sichuan. Under SSP5-8.5, it shifted northwestward twice and then northeastward to Gansu. Overall, both models predicted northward and westward centroid migration under future scenarios, particularly under SSP5-8.5. Notably, the China model under low-emission SSP1-2.6 predicted a southeastward shift to Chongqing, suggesting that milder climate change may produce less consistent directional shifts compared to the strong northwestward trends under SSP5-8.5. The China model also showed a more pronounced westward shift than the global model under SSP5-8.5.

## 4. Discussion

### 4.1. Spatial Extent of Occurrence Data vs. Algorithm Choice

As one of the most widely applied ensemble species distribution modeling platforms, Biomod2 has the major advantage of reducing uncertainty associated with individual models by integrating multiple algorithms [11]. However, the comparison results in this study demonstrated that the spatial extent of occurrence data introduced greater variation in model predictions than differences among modeling algorithms. This finding suggests that while considerable efforts are often devoted to algorithm selection, parameter optimization, and model tuning, a more fundamental issue may be overlooked: whether the spatial extent of occurrence records used for model calibration appropriately matches the scale of the research objective and ecological processes. This issue is particularly important for highly dispersive and recently introduced invasive pests such as *Spodoptera frugiperda* [23,36]. Our results indicated that models based on global occurrence data generally outperformed those based on China-only occurrence data. The ensemble model constructed using global occurrence data showed excellent predictive performance, exceeding that of all individual models included in the ensemble, and therefore provides a reliable basis for future assessments of the potential distribution of *S. frugiperda*. In contrast, the ensemble model based on China occurrence data showed only acceptable performance, with evaluation metrics even lower than those of several individual models, including XGBOOST, MaxEnt, MaxNet, and GBM. Therefore, although models of China based on China occurrence data can provide useful information for local-scale habitat suitability assessments, their application for estimating the overall potential distribution range of invasive species remains limited.

Another noteworthy finding is that ensemble averaging did not consistently outperform the best single algorithm. In the China model, the ensemble TSS (0.690) was markedly lower than that of XGBoost (0.787), whereas for the global model, the ensemble performance (0.851) was comparable to XGBoost (0.858). This discrepancy suggests that when occurrence data are geographically restricted and ecologically truncated, even high-performing algorithms such as XGBoost may capture local patterns more effectively than an average of multiple models, which can be diluted by less accurate algorithms [37]. Therefore, the advantage of ensemble modeling—reducing algorithm-specific uncertainty—is not unconditional; it depends on the quality, completeness, and environmental representativeness of the input data. This reinforces our central conclusion that, within the context of our study design, the spatial extent of occurrence data is a major determinant of model outcomes, at least as influential as the choice of algorithm.

### 4.2. Environmental Variable Contributions and Model Comparability

The contribution of environmental variables further revealed differences between the two modeling approaches. The global occurrence-based ensemble model exhibited a more balanced response to environmental variables, with more predictors retained during model construction and relatively even contributions among variables. This suggests that global-scale occurrence data may better capture the potential ecological niche of *S. frugiperda* [38,39]. In contrast, the China occurrence-based model showed a strong dependence on a single predictor, with Bio06 accounting for the majority of model importance. However, the high contribution of Bio06 was not simply a consequence of model bias but was also influenced by variable selection and multicollinearity among environmental predictors. Correlation analyses based on both global and China environmental datasets indicated strong correlations between Bio06 and both Bio08 and Bio09. In the China occurrence-based model, Bio08 and Bio09 were excluded because their contributions were lower than that of Bio06, resulting in Bio06 becoming the dominant climatic predictor and consequently receiving a disproportionately high contribution during model calibration [21]. Therefore, this pattern reflects not only the climatic response of *S. frugiperda*, but also the influence of restricted environmental gradients associated with regional occurrence datasets on variable selection and model interpretation.

A critical caveat must be emphasized when interpreting the comparative performance between the global and China models. The two ensemble models were calibrated with different sets of environmental predictors (13 variables retained for the global model versus 10 for the China model), which inevitably undermines the strict comparability of their variable contributions and prediction magnitudes [40]. Higher AUC and TSS values for the global model primarily indicate stronger discriminative ability—i.e., the capacity to distinguish presence from absence across a broader environmental gradient—rather than unequivocal predictive accuracy for the specific geographic region of China [41,42]. Likewise, a wider predicted suitable area does not automatically reflect greater reliability; it may partly arise from the larger calibration extent and more diverse environmental contrasts, which can inflate model sensitivity and broaden predicted envelopes without necessarily improving local-scale realism [32,43]. Therefore, the global model should be viewed as providing a more comprehensive characterization of the species’ fundamental niche rather than a definitively “truer” forecast for China. For practical management in China, predictions derived from the global model remain subject to extrapolation uncertainty, and their spatial outputs should be validated with independent occurrence data or field surveys whenever possible [44,45]. Consequently, our conclusion that spatial extent exerts a greater influence than algorithm selection should be interpreted with caution, as the comparison is confounded by the use of different variable sets. We recommend that future studies comparing occurrence data extents control for predictor composition to isolate the effect of data extent from that of variable selection.

### 4.3. Spatial Consistency of Core Suitable Habitats and Management Implications

Furthermore, this study demonstrated a high degree of consistency between the global occurrence-based model and the China occurrence-based model in identifying the core areas of highly suitable habitats for *Spodoptera frugiperda*. Both models consistently predicted that the major highly suitable regions were located in southern China and the Huang-Huai Plain. This spatial agreement was unlikely to be coincidental, but rather reflected the relatively high stability of the core component of the species’ fundamental niche that represents conditions closest to ecological optimum. Regardless of the spatial extent of occurrence data used for model calibration, the physiological tolerance ranges of *S. frugiperda* to key environmental factors are constrained by its biological characteristics, and therefore the core suitable regions are unlikely to undergo fundamental changes simply due to expansion of the training data extent [24]. Accordingly, highly suitable habitats can be considered one of the most robust prediction outputs across different modeling strategies and represent relatively reliable spatial information with lower uncertainty for practical monitoring and management. This finding is consistent with the conclusion of Elith et al. that prediction consistency among models generally increases with increasing suitability levels [46]. Notably, the Huang-Huai Plain is also one of the major maize-producing regions in China, where important host crops of *S. frugiperda* are extensively cultivated. The abundant and continuous distribution of host resources provides stable food availability for this pest, which may partly explain the ecological mechanisms underlying the concentration of highly suitable habitats in this region [9,47]. Study identification of these core areas is broadly consistent with previous MaxEnt-based predictions for *S. frugiperda* in China, which also identified southern and eastern China as highly suitable regions [25].

From a management perspective, this region should be prioritized for year-round monitoring, including fixed trapping networks and regular field surveys during the growing season. Early warning systems should also be extended to the northwestern margins of the current suitable areas, particularly in northern Henan, western Shandong, and southern Hebei, where future northward expansion is predicted under SSP5-8.5. Management strategies should be tiered according to emission scenarios: under moderate warming (SSP2-4.5), strengthened monitoring in currently suitable areas may suffice, whereas under high warming (SSP5-8.5), proactive measures—including resistant maize varieties and coordinated regional control—should be considered in preparation for range expansion. However, the spatial robustness of highly suitable habitats does not necessarily indicate temporal stability.

### 4.4. Future Dynamics: Nonlinear Responses and Centroid Migration

The results showed that, for both the global and China occurrence-based models, the total suitable habitat area under all three future climate scenarios in the 2050s was greater than that under the current climate scenario (Table 3). However, this net expansion does not imply a simple linear process. Instead, spatial change analysis (Figure 5) revealed that the expansion was accompanied by habitat losses in some currently suitable regions, particularly in western and northern China, indicating a simultaneous process of local contraction and expansion—a pattern of ‘redistribution’ rather than monotonic range enlargement [2]. This pattern highlights the nonlinear and heterogeneous effects of climate change on species distributions: climate change does not simply ‘expand’ suitable areas uniformly; rather, it drives the reorganization of ecological conditions through interactions among temperature, precipitation, and extreme climatic events [20]. The temporary spatial losses observed in some regions during the 2050s may indicate that altered temperature–precipitation combinations temporarily render some currently suitable areas unsuitable, even while new areas become available. With continued climate change from the 2070s to the 2090s, high-latitude and high-elevation regions may become increasingly suitable, leading to further northward and westward range expansion, particularly under SSP5-8.5. Climate change is not simply a linear process of “warming leading to expansion”; rather, it involves the reorganization of ecological conditions driven by interactions among temperature, precipitation, and extreme climatic events [48]. The temporary reduction in suitable habitats during the 2050s may indicate that, during the early stages of climate change, some currently suitable regions may become temporarily unsuitable due to altered temperature–precipitation combinations. For example, increasing temperatures may exceed the optimal thermal thresholds of *S. frugiperda* populations in some regions, while altered precipitation patterns may reduce water availability during the growing season, thereby disrupting previously favorable conditions [17]. With continued climate change from the 2070s to the 2090s, temperature constraints may gradually become less limiting, allowing high-latitude and high-elevation regions previously restricted by insufficient thermal conditions to become increasingly suitable. Consequently, suitable habitat areas may recover and exceed current levels. This pattern of “spatial robustness but temporal nonlinearity” indicates that the stability of *S. frugiperda* suitable habitats is dimension-dependent. At the spatial scale, the identification of core suitable habitats is relatively insensitive to the spatial extent of occurrence data; however, at the temporal scale, future climate change may substantially reshape habitat boundaries and expansion patterns. Therefore, future management strategies should not rely solely on the assumption of continuous long-term expansion but should incorporate dynamic habitat changes across different temporal windows [49].

The spatial robustness of highly suitable habitats does not guarantee equivalent reliability of the China model in predicting the overall potential distribution of *S. frugiperda*. China, as one of the terminal regions of the global invasion, was first colonized only in 2019 and is still undergoing range expansion. Consequently, the occurrence records within China have likely not yet reached ecological equilibrium with local climates, limiting the ability of the China model to fully characterize the species’ realized niche [50]. Given that China records are a geographic subset of the global dataset, The environmental space represented by Chinese occurrence records constitutes only a limited subset of the global environmental space potentially suitable for *S. frugiperda*. Moreover, these records may predominantly represent the peripheral portion of the species’ environmental tolerance range rather than its central niche space. Therefore, models calibrated using such regional occurrence data may suffer from incomplete environmental gradients and niche truncation, which can constrain the accuracy of ecological niche parameter estimation and reduce the reliability of extrapolation to unsampled regions [51].

This geographic limitation also influenced centroid migration patterns. Under current climate, the global model placed the centroid in Hebei Province, whereas the China model located it in Henan Province—reflecting their different characterizations of the spatial center of suitable habitats. The global model, incorporating broader environmental gradients, predicted a more northern centroid; the China model, constrained by niche truncation, yielded a more southern one. Despite these differences, both models showed consistent northwestward centroid shifts under future scenarios, with displacement magnitudes increasing with emission intensity. Notably, the China model exhibited a stronger westward shift than the global model, likely due to its more southern starting position and greater sensitivity to temperature changes in western Chin [52]. This suggests that regional models may detect the direction but amplify the magnitude of centroid shifts due to niche truncation, so migration distances should be interpreted conservatively. These cross-model consistencies suggest that the overall direction of centroid migration represents a relatively robust indicator of species responses to climate change and is less affected by the spatial extent of occurrence data used for model calibration. However, interpretation of migration magnitude requires careful consideration of the geographic background and ecological coverage of the training data. This finding provides an important methodological implication for future species distribution modeling studies: when different modeling strategies produce divergent predictions in terms of suitable habitat area or suitability categories, centroid migration direction may serve as a more robust comparative metric than changes in habitat extent.

### 4.5. Strengths, Limitations, and Rationale for Ensemble Approach

The major strength of this study is its systematic evaluation of how occurrence data extent influences Biomod2-based SDM predictions and its mechanistic explanation of global vs. regional model differences from invasion endpoints and niche truncation. The inclusion of centroid migration analysis as a complementary comparative metric also provides methodological guidance for selecting occurrence data scales and interpreting prediction uncertainty in invasive species modeling. Nevertheless, several limitations should be acknowledged. First, occurrence records were mainly obtained from GBIF (https://doi.org/10.15468/dl.mvr2h5, accessed on 20 June 2026), the literature, and field surveys; despite spatial filtering and thinning, biases from uneven sampling intensity and geographically biased survey efforts may persist [53]. Second, this study focused on climatic and topographic variables, while host plant distributions, biotic interactions, agricultural practices, and human activities were not considered [54], though these may substantially influence *S. frugiperda* establishment and spread. Third, SDM-based future projections assume species equilibrium with environmental conditions—an assumption violated here, as *S. frugiperda* invaded China only recently (2019) and is still expanding. The temporal mismatch between post-2019 occurrence records and long-term average climate data (WorldClim 2.1, 1970–2000) adds further uncertainty. Fourth, future projections used only one GCM (BCC-CSM2-MR); a multi-model ensemble would better capture climate uncertainty, though our comparative conclusions are unlikely to change. Fifth, no temporally independent occurrence data exist for external validation due to the recent invasion; our spatial cross-validation provides a standard alternative, but independent validation would strengthen confidence in model transferability. Therefore, the equilibrium assumption may introduce additional uncertainty into future distribution projections. Critically, however, this disequilibrium does not weaken our conclusions—it directly supports our finding that regional data from ongoing invasions are subject to niche truncation (as evidenced by the dominance of Bio06 in the China model), reinforcing the value of global data for comprehensive invasion risk assessment.

Fourth, future projections were based on a single GCM (BCC-CSM2-MR). While this model has demonstrated good performance in simulating temperature and precipitation over China and was selected for its regional relevance, we acknowledge that a multi-GCM ensemble would better characterize the uncertainty range of future climate projections. However, given the comparative nature of our core findings—namely, the consistent superiority of the global model over the China model—we consider it unlikely that the choice of GCM would alter our main conclusions.

Although the ensemble model for the China dataset (TSS = 0.690) underperformed the best individual algorithm, XGBoost (TSS = 0.787), we retained the ensemble approach for the final predictions. This decision was based on three considerations. First, ensemble modeling reduces algorithm-specific uncertainty—the final prediction is an average of six different algorithms (XGBoost, MaxEnt, MaxNet, GBM, GLM, MARS), each with distinct assumptions and data requirements. This averaging effect lowers prediction variance and enhances robustness, which is particularly important for projections under future climate scenarios where extrapolation is required [55]. Second, no single algorithm is universally optimal; the best-performing algorithm in one dataset or region may not perform equally well when transferred to other geographic or temporal conditions. Ensemble models offer a safeguard against over-reliance on a single modeling approach [2]. Third, the slightly lower TSS of the China ensemble model reflects not a flaw of ensemble averaging, but rather the limited quality of the training data—the regional occurrence records are subject to niche truncation and incomplete environmental representation. Under such conditions, even a high-performing algorithm like XGBoost may capitalize on spurious correlations that do not generalize well. Thus, the ensemble model—despite its marginally lower evaluation metrics—provides a more conservative and balanced representation of the species’ potential distribution, making it preferable for informing management decisions.

Future studies should integrate pest dispersal capacity, migration behavior, and agricultural landscape characteristics into predictive frameworks. Coupling climate-based species distribution models with mechanistic population or process-based models may further improve the ecological realism and predictive accuracy of future invasion risk assessments [56].

## 5. Conclusions

This study demonstrates that the spatial extent of occurrence data is a critical determinant of species distribution model predictions. Models calibrated with global data exhibited superior predictive performance and provided a broader characterization of the species’ environmental niche compared to China-only models, which suffered from niche truncation and strong dependence on a single climatic variable. Despite these differences, both models consistently identified southern China and the Huang-Huai Plain as core high-suitability areas, highlighting the spatial robustness of these regions. Future projections revealed a northward and westward expansion of suitable habitats, particularly under high-emission scenarios, though the China model predicted a substantially stronger westward shift, likely due to regional niche truncation. Under low-emission scenarios, the China model showed more variable directional shifts, suggesting that milder climate change may produce less consistent migration trends. Overall, the global model provides a broader environmental envelope that reduces the risk of niche truncation when assessing potential range expansion, whereas the China model remains valuable for identifying localized high-risk areas, but its conservative predictions should be interpreted with caution given the ongoing invasion process. These findings underscore that the choice of occurrence data extent must align with the specific research question and geographic context; neither global nor regional models should be regarded as universally superior.

## Figures and Tables

**Table 1:** Environmental variables selected for species distribution modeling.

Global Model	China Model
aspect	aspect
bio02	bio02
bio03	bio04
bio07	bio06
bio08	bio10
bio09	bio14
bio15	bio15
bio16	elev
bio17	gm_ve
bio19	slope
elev	
gm_ve	
slope	

**Table 2:** Mean evaluation metrics of individual models and ensemble models based on global and China occurrence datasets (values reported are calibration (training-set) metrics, indicating model fit to the data used for parameter estimation; values rounded to three decimal places).

Region	World			China		
Calibration Metrics	KAPPA	TSS	ROC	KAPPA	TSS	ROC
XGBOOST	0.824	0.858	0.976	0.712	0.787	0.951
SRE	0.441	0.434	0.717	0.385	0.427	0.713
RF	1.000	1.000	1.000	1.000	1.000	1.000
MaxNet	0.758	0.786	0.957	0.573	0.690	0.912
MaxEnt	0.745	0.753	0.947	0.602	0.693	0.917
MARS	0.700	0.732	0.933	0.559	0.666	0.900
GLM	0.685	0.697	0.933	0.550	0.673	0.900
GBM	0.687	0.716	0.937	0.569	0.686	0.906
GAM	0.585	0.604	0.891	0.520	0.652	0.877
FDA	0.573	0.617	0.873	0.504	0.654	0.870
CTA	0.685	0.655	0.893	0.557	0.642	0.885
ANN	0.480	0.520	0.770	0.492	0.644	0.826
EM	0.728	0.851	0.977	0.481	0.690	0.905

Footnote: Calibration metrics may overestimate true predictive performance due to overfitting, particularly for flexible algorithms such as RF. Model selection for ensemble construction was based on cross-validated performance, not on these calibration values. RF was excluded due to its known propensity for overfitting and poor transferability; Values represent model fit to training data; model selection for ensemble construction was based on cross-validated performance.

**Table 3:** Changes in suitable habitat area (×10^3^ km^2^) of *Spodoptera frugiperda* under different future climate scenarios and projection periods.

ID	World			China		
	Low	Moderate	High	Low	Moderate	High
current	797.20	1485.76	1179.34	1356.15	924.90	128.04
SSP1-2.6_2050s	1491.44	2089.57	851.13	1484.10	1055.64	566.82
SSP1-2.6_2070s	1465.23	2167.66	775.24	1618.45	988.40	473.09
SSP1-2.6_2090s	1526.23	2188.84	771.02	1521.41	999.46	554.39
SSP2-4.5_2050s	182.043	2189.50	812.20	1920.76	1034.38	649.41
SSP2-4.5_2070s	1946.72	2172.40	899.65	1973.96	1068.82	732.53
SSP2-4.5_2090s	2178.56	2195.92	881.67	2070.61	1094.69	846.27
SSP5-8.5_2050s	2004.84	2128.66	934.62	1857.52	1068.42	624.44
SSP5-8.5_2070s	2498.19	2213.58	971.75	2261.74	1122.99	778.92
SSP5-8.5_2090s	2940.12	2193.06	1049.91	2339.64	1238.32	889.06

## Data Availability

For manuscripts that are accepted, all authors agree to make data and materials available to third-party academic researchers upon reasonable request.

## Supplementary Materials

The following supporting information can be downloaded at https://www.mdpi.com/article/10.3390/insects17080843/s1: Figure S1: Heatmap of correlations among environmental factors; Table S1: All environmental variables used in this study; Table S2: Contribution of environmental factors to the model.
