# Supplementary material for: Effects of Different Spatial Extents of Occurrence Data on Biomod2-Based Species Distribution Modeling and Prediction: A Case Study of the Potential Distribution of Spodoptera frugiperda in China
Source: Insects. 2026 Aug 14;17(8):843. doi: 10.3390/insects17080843 (PMC13513867; doi:10.3390/insects17080843)
Supplement: Supplementary file 1 [file insects-17-00843-s001.zip › insects-4482602-supplementary.pdf]

Figure S1 Heatmap of correlations among environmental factors. (The former figure shows the correlation heatmap of environmental factors for China, and the latter shows the correlation heatmap of environmental factors globally.)

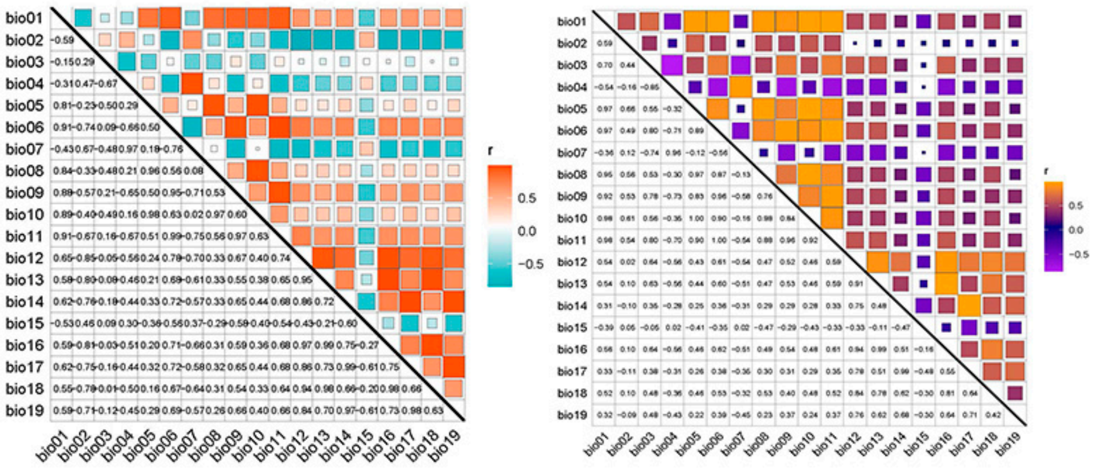

Table S1. All environmental variables used in this study.

Abbreviation	Description
BIO1	Annual mean temperature(℃)
BIO2	Mean diurnal range (monthly mean (max temp- min temp))(℃)
BIO3	Isothermally (BIO2/BIO7)×100
BIO4	Temperature seasonality (standard deviation×100)(C of V)
BIO5	Max temperature of the warmest month(℃)
BIO6	Min temperature of the coldest month(℃)
BIO7	Temperature annual range(BIO5-BIO6)(℃)
BIO8	Mean temperature of the wettest quarter(℃)
BIO9	Mean temperature of the driest quarter(℃)
BIO10	Mean temperature of the warmest quarter(℃)
BIO11	Mean temperature of the coldest quarter(℃)
BIO12	Annual precipitation(mm)
BIO13	Precipitation of the wettest month(mm)
BIO14	Precipitation of the driest month(mm)
BIO15	Precipitation seasonality(C of V)
BIO16	Precipitation of the wettest quarter(mm)
BIO17	Precipitation of the driest quarter(mm)
BIO18	Precipitation of the warmest quarter(mm)
BIO19	Precipitation of the coldest quarter(mm)
gm_ve_v2	Percentage of vegetation coverage

Table S2 Contribution of environmental factors to the model

World		China	
name	Importance (%)	name	Importance (%)
aspect	0.0015	aspect	0.03%
bio02	0.0656	bio02	0.69%
bio03	0.1359	bio04	2.89%
bio07	0.043	bio06	84.38%
bio08	0.3166	bio10	0.77%
bio09	0.139	bio14	0.76%
bio15	0.023	bio15	0.53%
bio16	0.2123	elev	1.24%
bio17	0.0201	gm_ve	8.69%
bio19	0.0028	slope	0.02%
elev	0.0281		
gm_ve	0.01		
slope	0.0021		
